# Supplementary material for: Identification of photoperiod-induced specific miRNAs in the adrenal glands of Sunite sheep (Ovis aries)
Source: Front Vet Sci. 2022 Jul 22;9:888207. doi: 10.3389/fvets.2022.888207 (PMC9354845; doi:10.3389/fvets.2022.888207)
Supplement: Supplementary file 1 [file Data_Sheet_1.ZIP › Supplementary file/miRDeep2 file.zip!/miRDeep2 file/Know_Structure/SP42A2/oar-let-7a.pdf]

miRBase precursor	: oar-let-7a
Total read count	: 366334
oar-let-7a read count	: 363797
remaining reads	: 2537

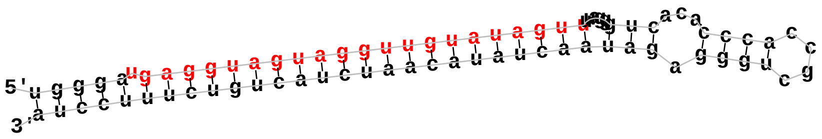

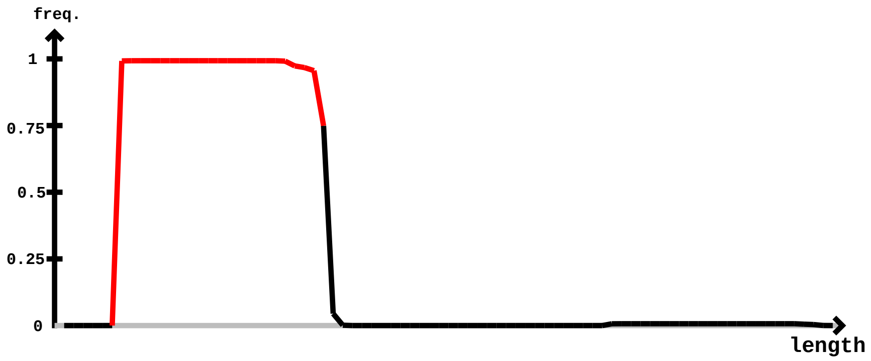

oar-let-7a

```
5'- ugggaugagguaguagguuguauaguuuuagggucacacccaccgcugggagauaacuauacaaucuacugucuuuccua   -3'   exp
    (((((.(((((((((((((((((((((.....(((...((((....)))).)))))))))))))))))))))))))))))   reads   mm   sample
    ....augagguaguagguuguau.........................................................   1       0    seq
    ....Uugagguaguagguuguauagu......................................................   6       1    seq
    ....augagguaguagguuguauagu......................................................   3       0    seq
    ....Uugagguaguagguuguauaguu.....................................................   8       1    seq
    ....augagguaguagguuguauaguu.....................................................   11      0    seq
    ....augagguaguagguuguauaguuu....................................................   2       0    seq
    .....ugagguUguagguugua..........................................................   1       1    seq
    .....ugaggCaguagguugua..........................................................   1       1    seq
    .....ugaAguaguagguugua..........................................................   6       1    seq
    .....ugagguagGagguugua..........................................................   60      1    seq
    .....ugagguaguaAguugua..........................................................   5       1    seq
    .....ugagguaguagguuUua..........................................................   1       1    seq
    .....ugagguaguagguugua..........................................................   507     0    seq
    .....ugagguaguagguuUuau.........................................................   15      1    seq
    .....ugagguaguagguuguau.........................................................   5887    0    seq
    .....ugagguUguagguuguau.........................................................   9       1    seq
    .....ugaAguaguagguuguau.........................................................   50      1    seq
    .....ugagguagGagguuguau.........................................................   555     1    seq
    .....ugagguaguaAguuguau.........................................................   35      1    seq
    .....ugagguaguagguuguaua........................................................   2056    0    seq
    .....ugagguagGagguuguaua........................................................   181     1    seq
    .....ugagguaguagguuguauag.......................................................   3644    0    seq
    .....ugagguagGagguuguauag.......................................................   614     1    seq
    .....ugagguagGagguuguauagu......................................................   8803    1    seq
    .....ugagguaguagguuguauagu......................................................   67015   0    seq
    .....ugagguaguagguuguauaguu.....................................................   233924  0    seq
    .....ugagguagGagguuguauaguu.....................................................   23805   1    seq
    .....ugagguaguagguuguauaguuu....................................................   15879   0    seq
    .....ugagguaguagguuguauaguuuA...................................................   68      1    seq
    .....ugagguaguagguuguauaguuuu...................................................   406     0    seq
    .....ugagguaguagguuguauaguuuua..................................................   4       0    seq
    ......gagguaguagguuguau.........................................................   3       0    seq
    ......gagguaguagguuguauagu......................................................   34      0    seq
    ......gagguagGagguuguauagu......................................................   8       1    seq
    ......gagguagGagguuguauaguu.....................................................   20      1    seq
```

oar-let-7a

```
ugggaugagguaguagguuguauaguuuuagggucacacccaccgcugggagauaacuauacaaucuacugucuuuccua
......gagguaguagguuguauaguu.....................................................    145    0    seq
......gagguaguagguuguauaguuu....................................................    15     0    seq
.......aggCaguagguuguauagu......................................................    1      1    seq
.......agguaguagguuguauagu......................................................    2      0    seq
........gguaguagguuguauaguu.....................................................    4      0    seq
........gguaguagguuguauaguuu....................................................    1      0    seq
.........guaguagguuguauaguu.....................................................    1      0    seq
..........uaguagguuguauaguuuu...................................................    1      0    seq
..................................................agauaacuauacaaucuacug.........    1      0    seq
...................................................gauaacuauacaaucuacugu........    2      0    seq
...................................................gauaacuauacaaucuacugucu......    1      0    seq
...................................................gauaacuauacaaucuacugucuu.....    1      0    seq
....................................................auaacuauacaaucuacugucuuu....    1      0    seq
.....................................................uaacuauacaaucuacugucu......    1      0    seq
.....................................................uaacuauacaaucuacugucuu.....    1      0    seq
.....................................................uaacuauacaaucuacugucuuu....    6      0    seq
........................................................cuauacaaucuacugucu......    6      0    seq
........................................................cuauacaaucuacugucuu.....    14     0    seq
........................................................cuauacaaucuacugucuuu....    599    0    seq
........................................................cuauacaauUuacugucuuu....    1      1    seq
........................................................cuauacaaucuacuguUuuu....    1      1    seq
........................................................cuauacaaucuacugucuuuc...    494    0    seq
........................................................cuauacaaucuacugucuuucc..    1251   0    seq
........................................................cuauacaaucuacugucuuuccu.    51     0    seq
.........................................................uauacaaucuacugucuuu....    4      0    seq
.........................................................uauacaaucuacugucuuA....    1      1    seq
.........................................................uauacaaucuacugucuuuc...    2      0    seq
.........................................................uauacaaucuacugucuuucc..    29     0    seq
.........................................................uauacaaucuacugucuuuccu.    49     0    seq
..........................................................auacaaucuacugucuuuc...    1      0    seq
..........................................................auacaaucuacugucuuucc..    4      0    seq
..........................................................auacaaucuacugucuuuccu.    14     0    seq
..........................................................auacaaucuacugucuuuccua    2      0    seq
```
